# Cyclic stretching-induced epithelial cell reorientation is driven by microtubule-modulated transverse extension during the relaxation phase

**DOI:** 10.1038/s41598-021-93987-y

**Published:** 2021-07-20

**Authors:** Jui-Chien Lien, Yu-li Wang

**Affiliations:** Department of Biomedical Engineering, Carnegie Mellon University, Pittsburgh, PA 15213 USA

**Keywords:** Biophysics, Cell biology

## Abstract

Many types of adherent cells are known to reorient upon uniaxial cyclic stretching perpendicularly to the direction of stretching to facilitate such important events as wound healing, angiogenesis, and morphogenesis. While this phenomenon has been documented for decades, the underlying mechanism remains poorly understood. Using an on-stage stretching device that allowed programmable stretching with synchronized imaging, we found that the reorientation of NRK epithelial cells took place primarily during the relaxation phase when cells underwent rapid global retraction followed by extension transverse to the direction of stretching. Inhibition of myosin II caused cells to orient along the direction of stretching, whereas disassembly of microtubules enhanced transverse reorientation. Our results indicate distinct roles of stretching and relaxation in cell reorientation and implicate a role of myosin II-dependent contraction via a microtubule-modulated mechanism. The importance of relaxation phase also explains the difference between the responses to cyclic and static stretching.

## Introduction

Increasing attention has been paid over past decades to the effect of external mechanical signals, such as stretching forces and substrate stiffness, on cellular behavior, including growth, shape, adhesion, polarity, and migration^1–4^. In particular, cyclic stretching takes place ubiquitously in the body, driven for example by beating of the heart, inflation of the lungs, or peristalsis of the gut. Many types of cells, including smooth muscle cells, endothelial cells, and epithelial cells, are exposed to cyclic stretching to affect both physiological and pathological events. For instance, elevated cyclic stretching was proposed to induce aortic valve calcification^5^, while proper stretching activities promote the differentiation of embryonic stem cells into muscle cells^6^.

As was first discovered four decades ago, cyclic stretching caused fibroblasts to reorient perpendicularly to the direction of stretching^7^. Subsequent studies confirmed that many different types of adherent cells, such as endothelial and epithelial cells, can realign similarly when exposed to uniaxial cyclic stretching^8–12^. It is generally believed that this stretch-induced realignment is of importance to such events as wound healing, angiogenesis, and morphogenesis^13–16^. The intriguing response, accompanied by reorganization of actomyosin contractility and realignment of the actin and microtubule cytoskeleton^17–21^, prompted various hypotheses based, for example, on the maintenance of tensional homeostasis or dissipation of stored elastic energy^9,22,23^. In addition to the contraction of actin cytoskeleton, microtubules may also play a regulatory role by stabilizing the cell shape and polarity^18,19,24^.

Stretching-induced transverse reorientation is sensitive to the amplitude, frequency, and waveform of stretching^9,10^. The response requires a minimal strain of around 3%^25^, above which the extent of reorientation shows a correlation with the magnitude of strain. Interestingly, static uniaxial stretching was found to cause cells to spread and migrate toward the direction of stretching^26,27^, in contrast to the transverse reorientation induced by cyclic stretching. Fibroblasts reorientation requires a minimal frequency of 0.01 Hz and saturates at 1 Hz, while smooth muscle cells show optimal realignment responses at 0.5 Hz^9,28^. Triangular, square, or asymmetric waveforms induce different extents of cell reorientation and stress fiber redistribution^10,29^, which suggests sensitivities to the slopes of stretching and/or duration of stretching/relaxation.

The above observations suggest that, to unveil the mechanism of cyclic stretching-induced cell reorientation, it is important to understand potentially differential responses during the stretching and relaxation phases. To this end, we have developed an on-stage cell stretcher based on a motorized microscope stage and an elastic, patternable polyacrylamide substrate. The system was programmed to allow synchronized image recording and cyclic stretching. Differences between consecutive images then revealed changes in cell shape during different stages of cyclic stretching and led us to a working model for transverse cell reorientation.

## Results

### Reorientation response of NRK-52E epithelial cells to cyclic stretching

**Figure 1:**
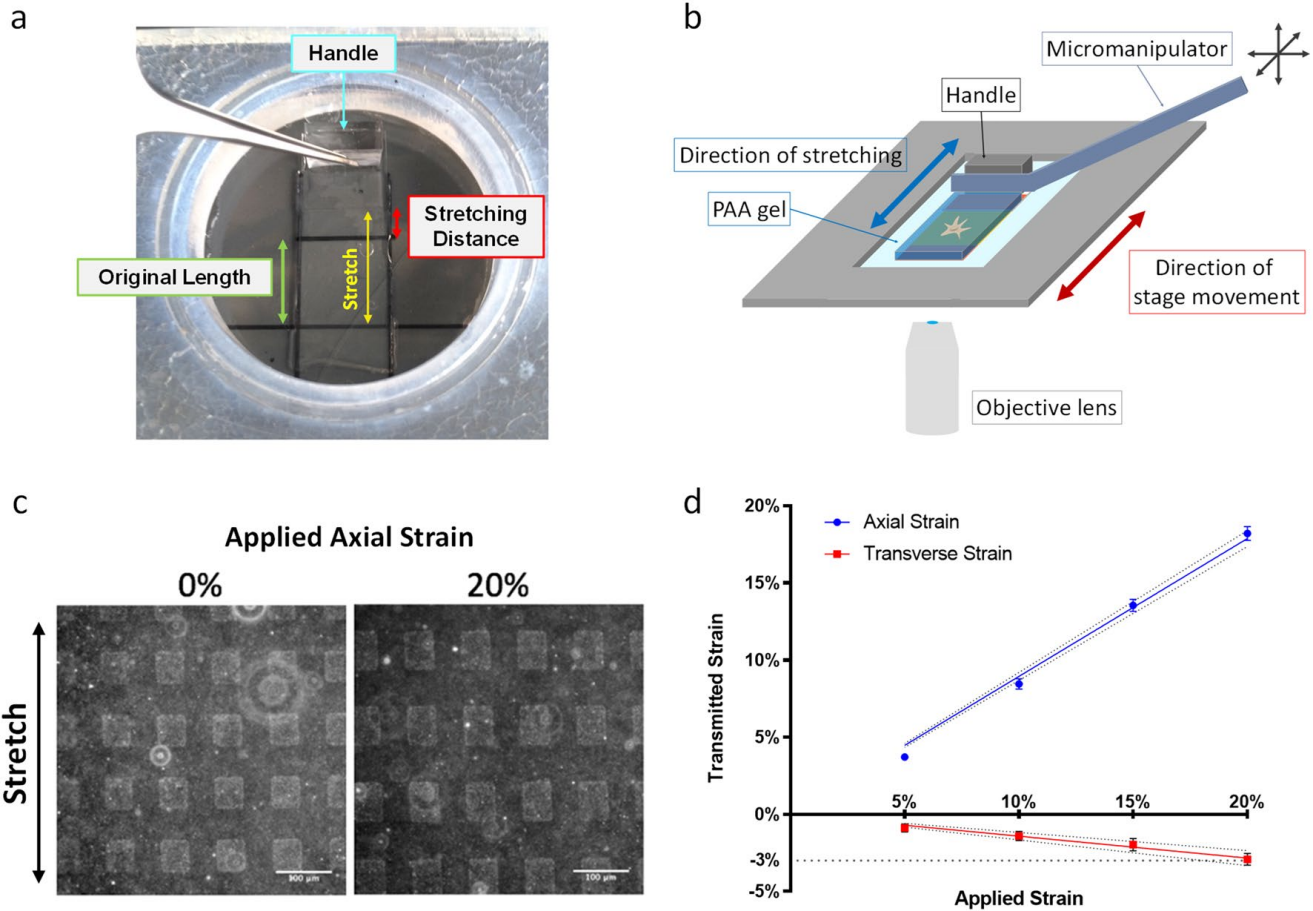
Cell stretcher built upon a microscope with a motorized stage. (**a**) The opposite ends of an elastic PAA gel are covalently bonded to a supporting coverslip and a handle, respectively. The stretcher is mounted on an acrylic holder to form a cell culture chamber and placed on the motorized microscope stage. (**b**) After locating the region of interest, the handle of the gel is anchored by a rod mounted on a micromanipulator. Back and forth movements of the stage cause cyclic stretching of the gel. (**c**) Micropatterned squares on the gel surface, 50 × 50 µm^2^ in size and 50 µm apart from each other, are revealed by the concentration of fluorescent beads underneath protein-coated regions during microcontact printing. (**d**) Axial and transverse strains of the gel, as measured by the deformation of the square pattern, vary linearly with calculated strains. However, axial strain is > 6 times larger than transverse strain (scale bar, 100 μm; n = 5, mean ± SEM).

NRK-52E cells, a rat kidney epithelial cell line that exhibits high circularity and large spreading area at steady state, were used for studying shape responses to cyclic stretching. A novel stretcher for real-time imaging was developed using a microscope with a motorized stage. Cells were cultured on an elastic polyacrylamide (PAA) substrate 400 μm in thickness. One end of the substrate was attached to the supporting coverslip underneath, which moved with the motorized stage. The other end was anchored with a rod, which pushed against a handle attached to the surface (Fig. 1a,b). A custom computer program allowed synchronized image acquisition at various times relative to the stretching cycle.

We used PAA gels with a Young's modulus of 20–30 kPa (prepared with 10% acrylamide and 0.3% bis-acrylamide), which is in the physiological range of tissue stiffness, as the substrate^30^. Micropatterns on the gel surface were used to indicate the applied strain (Fig. 1c; Supplementary Fig. 1f,i,k). The gel showed a nearly ideal elastic behavior when stretched by up to 20% at 0.5 Hz in a square waveform (Fig. 1d). Upon relaxation from 45 min of continuous cyclic stretching with 15% strain, the residual strain was < 0.72% along the direction of stretching (referred to as axial) and < 0.55% along a perpendicular direction (referred to as transverse) (Supplementary Fig. 2). The substrate remained intact following > 72 h of up to 18% of continuous cyclic stretching at 0.5 Hz. In addition, transverse strain remained below 16% of axial strain, suggesting that the strain was predominantly uniaxial (Fig. 1d).

**Figure 2:**
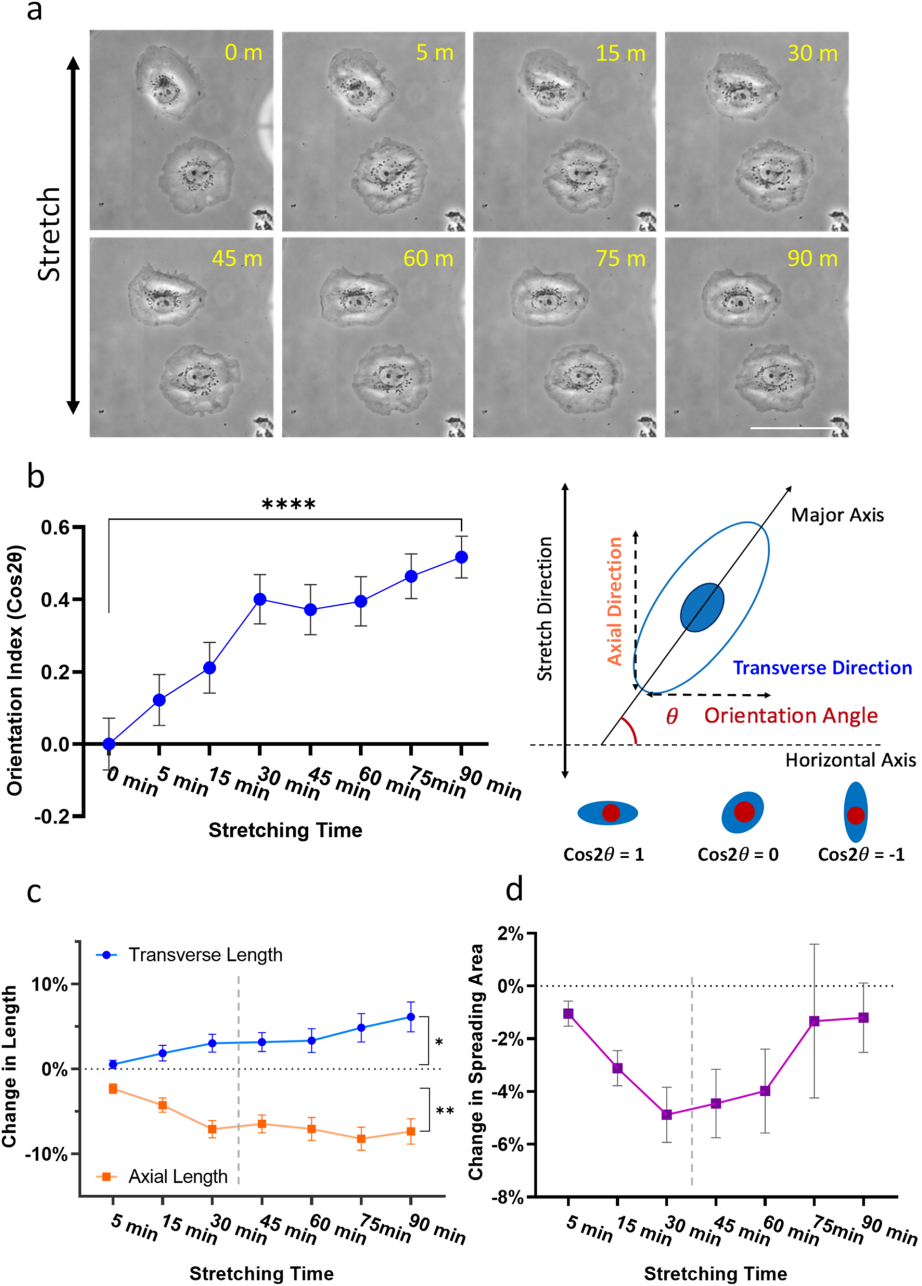
Changes in shape, orientation, and area of NRK-52E epithelial cells in response to uniaxial cyclic stretching. (**a**) Isolated NRK-52E cells show visible shape change and reorientation perpendicular to the direction of stretching over 90 min of stretching (scale bar, 100 μm). (**b**) Orientation index is calculated as *cos2θ* where *θ* is the angle between the major axis of the cell and the transverse direction. Transverse orientation, detectable as early as 5 min after the initiation of cyclic stretching, increases steadily over a period of 90 min. (**c**,**d**) Corresponding changes in cell length along axial and transverse directions, and in spreading area, are also observed during 90 min of cyclic stretching. Shape change is driven primarily by shortening along the axial direction during the first 30–45 min of cyclic stretching, in conjunction with a slow but continuous increase in transverse length and recovery in spreading area between 45 and 90 min (**c**,**d**). The two distinct phases before and after 30–45 min are indicated by vertical dashed lines (*p < 0.05; **p < 0.01; ****p < 0.0001; n = 82, mean ± SEM).

To avoid the complications of cell–cell mechanical interactions, we have limited the analysis to isolated cells. With 15% of cyclic strain at 0.4–0.5 Hz, cells started to show shape change as soon as 5 min and became oriented in a transverse direction within 30 min (Fig. 2a,b). By measuring the length along axial and transverse direction, we found that axial length decreased rapidly during the first 30 min, while transverse length steadily increased over 90 min (Fig. 2c). Consistent with the changes in length, spreading area decreased during the first 30 min then gradually recovered over the following 60 min (Fig. 2d). These observations indicated that stretching-induced reorientation involved a rapid axial shortening phase and a steady transverse elongation phase.

### Distinct shape responses during stretching and relaxation phases

**Figure 3:**
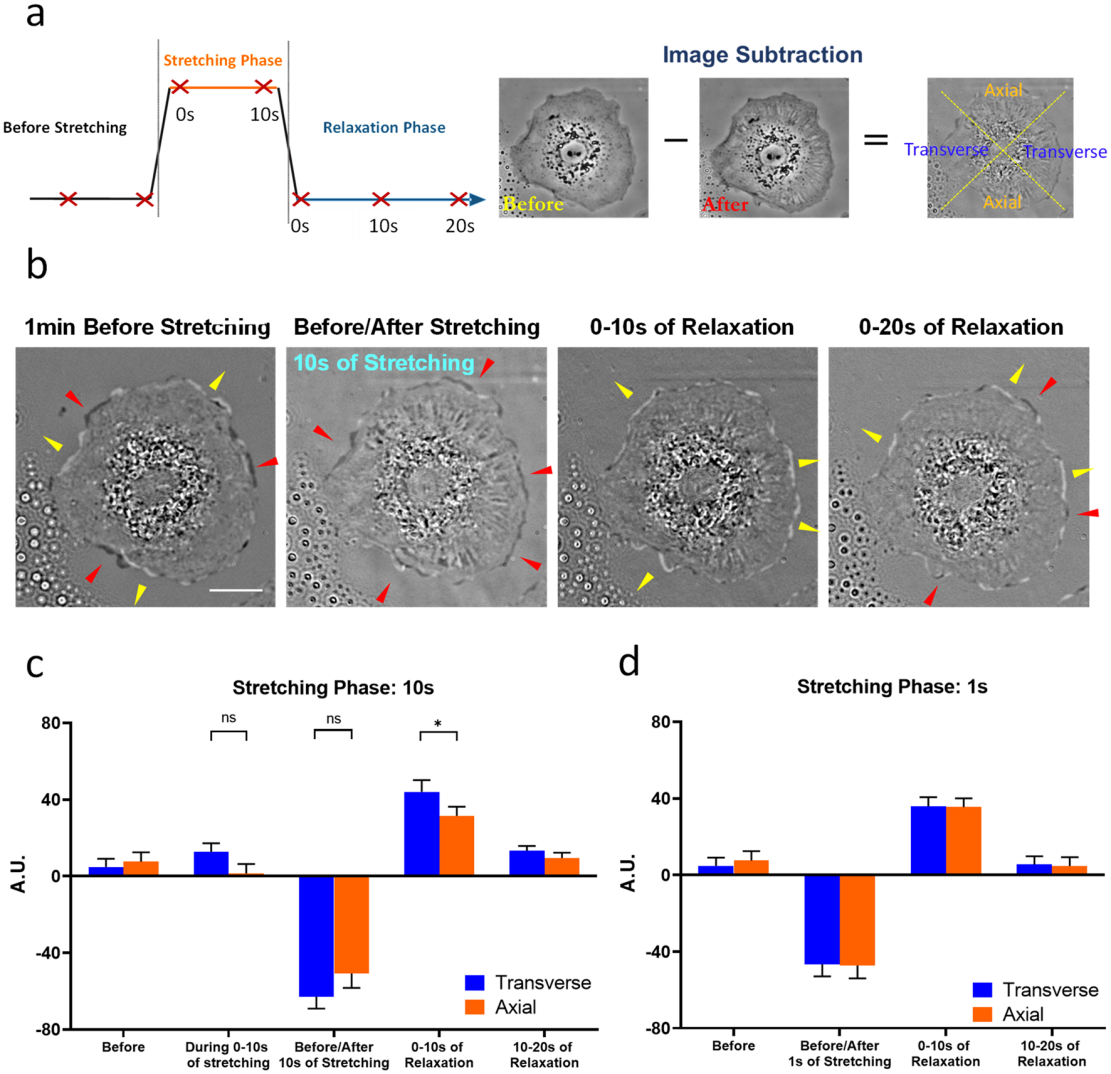
Distinct protrusive and retractive responses to a single pulse of stretching. (**a**) Images are collected before, during, and after stretching, as indicated by red crosses (left diagram). Shape changes are detected by difference imaging between consecutive images. Each cell is divided into two axial and two transverse quadrants, by drawing two diagonal lines at the centroid (right diagram). (**b**) Dark (red arrows) and bright (yellow arrows) regions in the difference image indicate retraction and protrusion, respectively. (**c**) Before and during 10 s of stretching, cells show only randomly distributed retractions and protrusions, resulting in little net change in peripheral area for transverse or axial quadrants. (**b**,**c**) Strong retractions occur immediately upon relaxation, while protrusions dominate during the following 10 s of relaxation. (**d**) Reducing the duration of stretching to 1 s has no significant effect on the extent of retraction but reduces the subsequent transverse extension (scale bar, 20 μm; n.s.* p* > 0.05, *p < 0.05; n = 20, mean ± SEM).

The above observations suggested that cellular responses to cyclic stretching may involve distinct protrusive/retractive responses in a spatially/temporally dependent manner, while the reorientation may represent a cumulative result of incremental shape changes. We, therefore, applied difference imaging as a sensitive means for detecting local changes in cell area (Fig. 3a). We first examined the response to a single pulse of stretching for 10 s by generating difference images before and after stretching, during stretching, and during post-stretching relaxation (Fig. 3b). To compare the activities along axial and transverse directions, we further divided each cell into two axial and two transverse quadrants (Fig. 3a).

Difference images taken during relaxation showed predominantly transverse protrusions (Fig. 3b), which were most prominent during the first 10 s after stretching then decreased rapidly (Fig. 3b,c). In contrast, difference images during stretching showed only baseline activities similar to those of unstretched cells, while difference images immediately before and after stretching showed prominently retractions along both axial and transverse directions (Fig. 3c), suggesting that retraction took place immediately after the release of stretching. Decreasing the duration of stretching from 10 to 1 s did not significantly decrease the extent of retraction but reduced the subsequent transverse protrusions and the difference in protrusive activities between transverse and axial directions (Fig. 3d).

**Figure 4:**
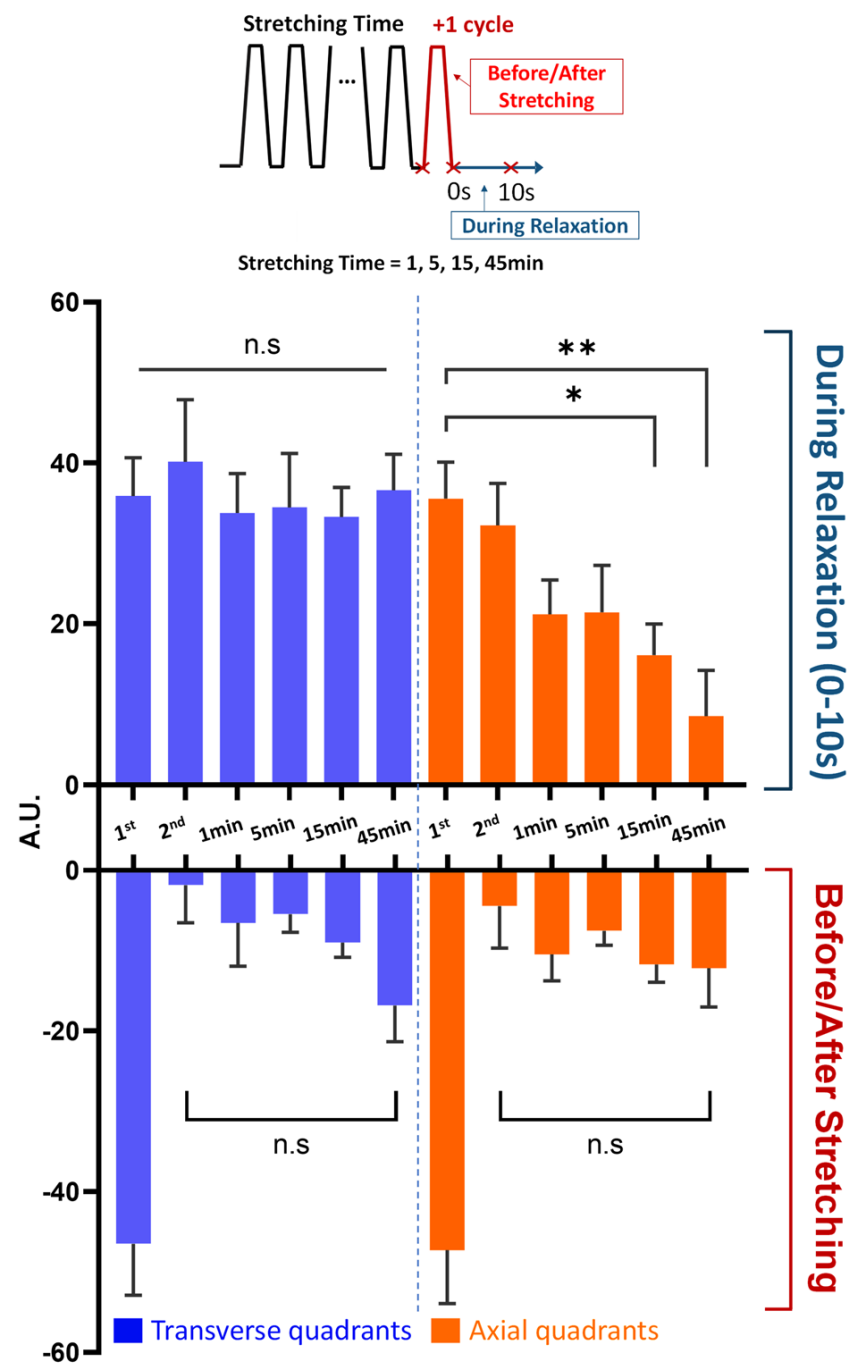
Differential axial and transverse responses following multiple cycles of stretching. Changes in peripheral areas are measured at the 1^st^ cycle, 2^nd^ cycle, and after various periods of cyclic stretching at 0.5 Hz as shown in the diagram, where the timing of image acquisition relative to the stretching cycles is indicated by red crosses. Local retractions or protrusions of different quadrants are then integrated and normalized against the total spreading area to reveal net extension (positive values) or retraction (negative values). Note that while the relaxation phase lasts for 1 s for cyclic stretching at 0.5 Hz, given the slow, persistent nature of extension, net extension was measured over a period of 10 s to improve the accuracy, after halting the cyclic stretching. Therefore, the actual extension during the relaxation phase is approximately 10% of the values shown in the upper graph. Net retraction takes place immediately after stretching (lower graph), while net extension takes place during subsequent 10 s of relaxation (upper graph). Moreover, net extension of axial quadrants decreases progressively with increasing cycles of stretching (top orange), while net extension of transverse quadrants remains constant (top blue). Retraction occurs similarly in all the quadrants, showing a strong response following a single cycle of stretching and a precipitous decrease with additional cycles of stretching (bottom graphs) (*p < 0.05; **p < 0.01; n = 16–20, mean ± SEM).

We then examined the response to stretching after applying cyclic stretching for up to 45 min at 0.5 Hz (Fig. 4). Interestingly, while protrusive activities of transverse quadrants were maintained following multiple cycles of stretching (Fig. 4, upper left blue bars), protrusions of axial quadrants decreased progressively (Fig. 4, upper right orange bars). In contrast, similar retraction responses were observed along axial and transverse quadrants immediately after the relaxation of stretching, showing strong retraction after a single cycle of stretching but much weaker retraction after additional cycles (Fig. 4). Together, these results explained cyclic stretching-induced reorientation as a cumulative consequence of persistent transverse protrusions coupled with diminishing axial protrusions.

### Functional roles of myosin II and microtubules in cyclic stretching-induced cell reorientation

**Figure 5:**
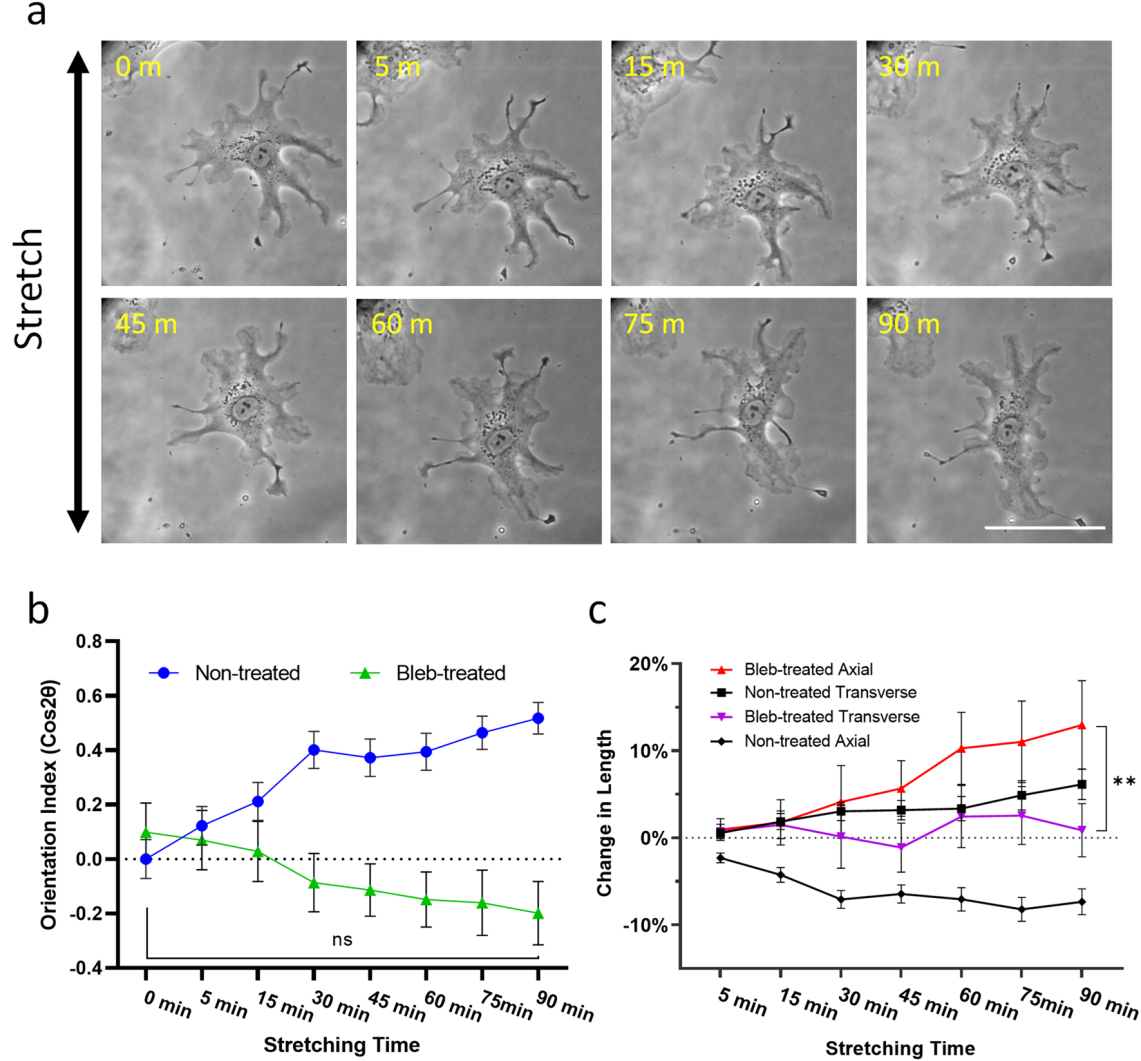
Effect of blebbistatin on cell reorientation and length change in response to cyclic stretching. (**a**) Myosin II activities are inhibited by treating cells with 50 μM blebbistatin for 30 min before applying cyclic stretching. Cells respond by elongating along axial direction (scale bar, 100 μm). (**b**) Unlike control cells (non-treated), blebbistatin-treated cells show weakly significant axial reorientation. (**c**) The response to blebbistatin involves an increase in axial length and an inhibition of transverse elongation (n.s. *p* > 0.05, **p < 0.01; n = 46, mean ± SEM).

Previous studies have implicated actomyosin contractility in cyclic stretching-induced cell reorientation^17,19^. By inhibiting myosin II with blebbistatin, we found that cells became highly branched while showing a weak orientation along the axial direction (Fig. 5a,b). In addition, both axial shortening and transverse elongation were suppressed as compared to untreated cells (Fig. 5c), suggesting that actomyosin contractility is directly or indirectly required for both activities.

**Figure 6:**
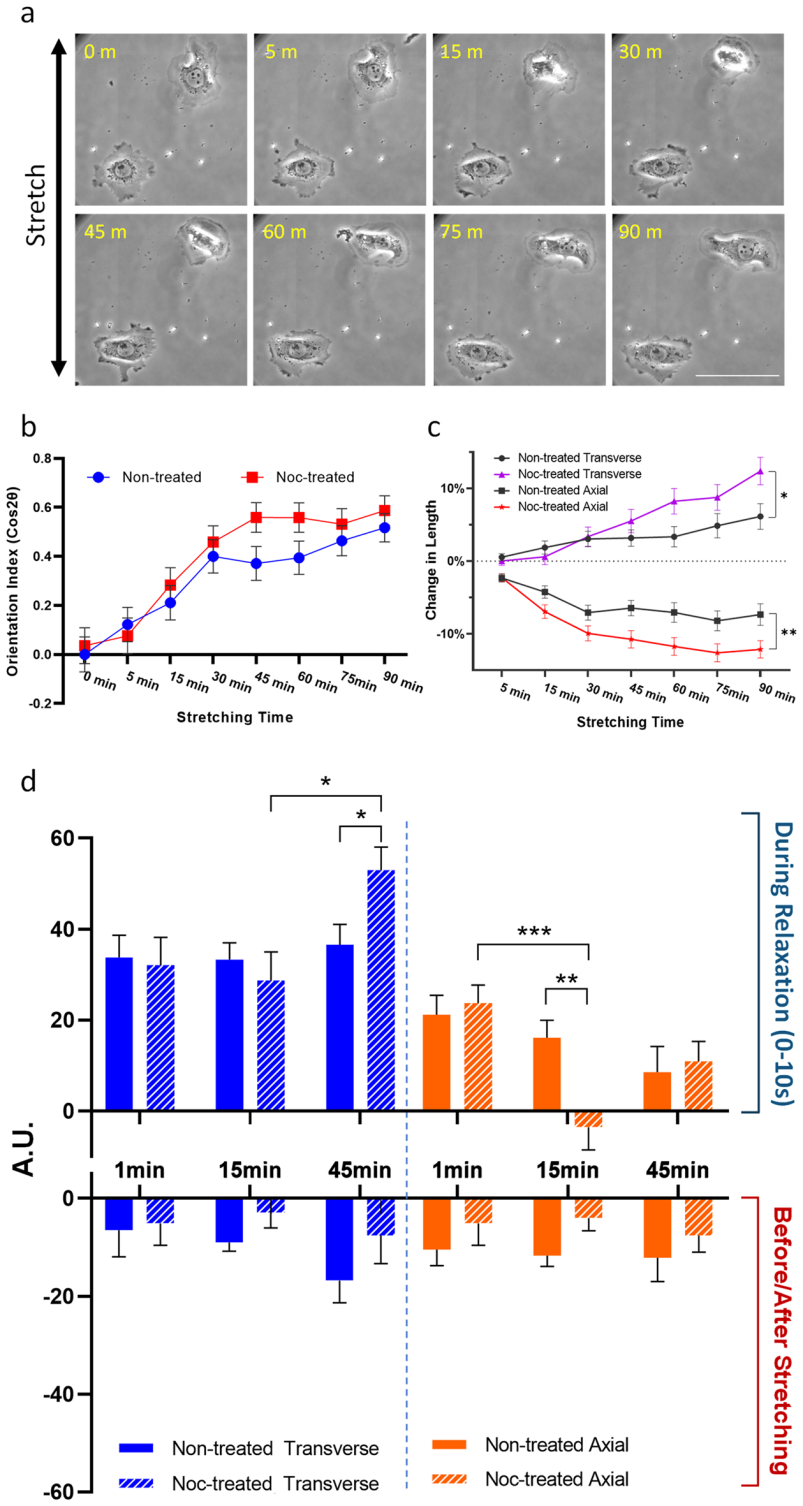
Effects of nocodazole on cell reorientation and length change in response to cyclic stretching. (**a**) Cells, treated with 5 μM nocodazole for 2 h before applying cyclic stretching, show more pronounced shape change and reorientation than control cells shown in Fig. 2. (**b**,**c**) Comparison of control and nocodazole treated cells for reorientation and length change suggests that the effects of nocodazole take place primarily after 30 min of cyclic stretching, as an increase in both transverse elongation and axial shortening (*p < 0.05; **p < 0.01; n = 98, mean ± SEM). Similar to the analysis in Fig. 4, changes of peripheral area are measured in nocodazole-treated and control cells. (**d**) Compared to the control (solid bars), nocodazole causes a transient inhibition of axial protrusion after 15 min of cyclic stretching (striped orange bar, upper graph), and a late stimulation of transverse protrusion at 45 min (striped blue bar, upper graph). Nocodazole treated cells also show net retraction immediately after stretching (lower graph), similar to control cells (*p < 0.05; **p < 0.01; ***p < 0.001; n = 16–20, mean ± SEM).

Since microtubules have been suggested to control cell shape and polarity^24,31,32^, we tested the effect of microtubule disassembly on the responses to cyclic stretching. As shown in Fig. 6a,b, treatment with nocodazole caused a visibly higher degree of reorientation than controls. Treatment with vincristine, a compound unrelated to nocodazole that also induces microtubule disassembly, caused a similar enhancement of cell reorientation (Supplementary Fig. 3a). Moreover, both axial shortening and transverse elongation reached a greater extent than control cells (Fig. 6c and Supplementary Fig. 3b), suggesting that microtubules play a role in tempering the response. Further analyses of the responses during the first 10 s of relaxation after various periods of cyclic stretching revealed that axial protrusion was inhibited transiently after 15 min of stretching (Fig. 6d), while transverse protrusion showed an increase after 45 min (Fig. 6d). These effects of nocodazole were consistent with the kinetics of length change as shown in Fig. 6c and implicated a microtubule-mediated mechanism that affects protrusive activities in a location-dependent manner.

Previous studies showed that microtubule disassembly can cause an increase in traction forces^33,34^, which may affect cyclic-stretch induced cell shape change. We first confirmed that a similar increase took place when NRK cells were treated with nocodazole (Supplementary Fig. 5a). Interestingly, combined treatment of nocodazole and blebbistatin caused NRK cells to reorient more prominently along the axial direction than treatment with blebbistatin alone, via a steady elongation in axial length and shortening in transverse length (Supplementary Fig. 4). Thus, inhibition of myosin II contractility caused the direction of reorientation to change from transverse to axial while disassembly of microtubules enhanced this response, suggesting that the reorientation effect of microtubules was mediated via myosin II and that microtubules modulated this myosin II dependent process.

## Discussion

Although cellular response to cyclic stretching has been studied for decades due to its physiological importance, the transverse reorientation as observed for adherent cells seems counterintuitive. A serious limitation has been the reliance on endpoint analysis in most studies, such that little is known about the dynamic events responsible for the reorientation. In the present study, we have developed a novel cell stretcher based on a motorized microscope stage that allows the recording of live cells in synchrony with cycles of stretching and relaxation. The study was further facilitated by the nearly ideal elastic property of PAA substrates, and by the use of difference imaging for detecting minute changes in cell shape in response to stretching or relaxation.

We found that the response to cyclic stretching took place via an early phase that lasted for 30–45 min, when the reorientation involved primarily net axial retraction, followed by a late phase when the reorientation involved primarily net transverse extension (Fig. 2c). This late phase of extension is similar to the lagged increase in traction forces along the transverse direction as reported previously^35^. In addition, the response to each stretching cycle involved no significant response during stretching, immediate retraction upon relaxation, and protrusion that persisted during the ensuing relaxation (Figs. 3c, 4). Retractive and protrusive activities showed distinct temporal and spatial patterns. Retraction was observed immediately upon relaxation along both axial and transverse directions. The extent was most prominent following the first cycle of stretching but decayed rapidly in subsequent cycles. Extension was detected during the subsequent period of relaxation. While extension also took place along both axial and transverse directions, the activity persisted along the transverse direction but decreased gradually along the axial direction over 30 min (Fig. 4). Together, these events explained not only the reorientation but also the transient decrease in cell area during the first 30 min (Fig. 2d).

The key role of relaxation phase in cyclic-stretching-induced reorientation provided a simple answer to the puzzling difference between the responses to cyclic and static stretching, where the reorientation was axial and too slow to be captured during the 1–10 s of stretching in the present cyclic regimen^1,26,27,36^. The requirement of relaxation for reorientation may also explain the dependence of the responses on the waveform of stretching; triangular waves were found to elicit much weaker responses than rectangular or trapezoid waves of a similar peak magnitude. We suspect that while rectangular and trapezoid waves contain discrete periods of relaxation to support transverse elongation, relaxation in triangular waves is gradual and total relaxation is too brief to allow much shape change^10,29^.

The present relationship between retraction and protrusion may be similar to that in the process of "retraction induced protrusion", where tail retraction was followed by frontal protrusion at the opposite end^32,37,38^. Similarly, symmetry breaking that initiates polarized cell migration typically starts with the formation of a retracting tail, followed by protrusion at the front^39,40^. All these activities may represent a common mechanism of retraction at one end triggering protrusion signals at a distal end. Supporting this hypothesis, we showed that the inhibition of myosin II with blebbistatin suppressed not only axial retraction but also transverse extension (Fig. 5), causing cells to orient along the axial direction, possibly as a passive response to stretching^17,19^.

Previous investigations of the role of microtubules in cell reorientation upon cyclic stretching have yielded conflicting results^19,41–43^. As microtubules are required for establishing cell polarity and directional cell migration^24,43,44^, we suspect that they may play a role in coordinating differential transverse and axial extensions, such that their disassembly may eliminate the difference between transverse and axial activities and inhibit cell reorientation. Unexpectedly, disassembly of microtubules caused an enhancement of cyclic stretching-induced reorientation through the enhancement of both axial retraction and transverse extension (Fig. 6). The observation may be explained by the role of microtubules to down-regulate myosin II dependent traction forces, which may be responsible for axial shortening possibly via the sequestration of GEF-H1 and deactivation of RhoA^45,46^. Axial contraction may in turn allow protrusions in regions distal to the axial ends as a downstream event to cause transverse elongation. Nocodazole-induced disassembly of microtubules may lead to an increase in axial contraction thereby promoting transverse protrusion. Conversely, inhibition of contractility with blebbistatin, with or without the disassembly of microtubules, may inhibit myosin II-dependent axial shortening, to suppress transverse elongation and allow protrusion to take place along the axial direction (Fig. 5, Supplementary Figs. 4 and 5).

A second, non-mutually exclusive possibility is that microtubules, as relatively rigid structures, may be aligned by stretching to generate anisotropic resistance to retraction^43^. Disassembly of microtubules may decrease this resistance and facilitate axial retraction. A third possibility is that retraction signals may be generated at axial ends then transported via microtubules to suppress extensions across the cell. Inhibition of microtubules would then cause retraction signals to accumulate at axial ends, enhancing axial retraction while allowing more extensions elsewhere (Supplementary Fig. 6). This hypothetical mechanism is therefore complementary to the local-excitation/global-inhibition (LEGI) mechanism proposed for polarized cell migration^47,48^, where microtubules were assumed to transport retraction signals away from the front to create a stable tail while protrusion signals were localized and self-amplified at the front^24^.

**Figure 7:**
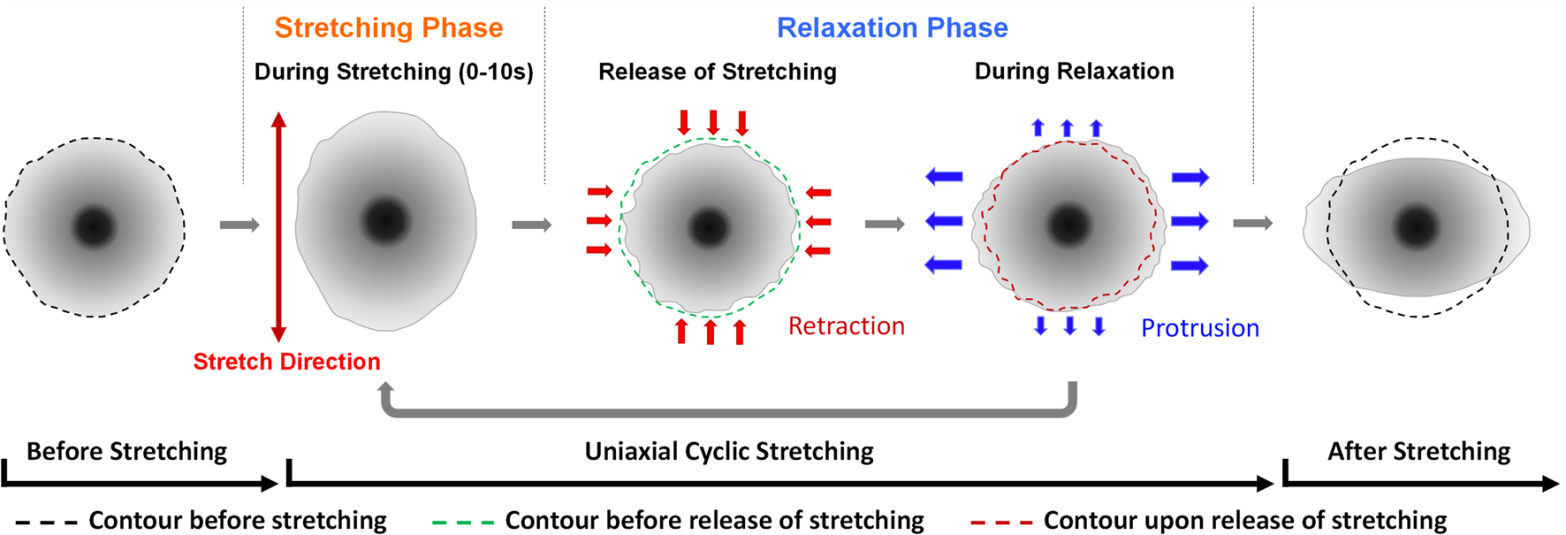
Summary of spatially and temporally dependent protrusion/retraction activities that lead to cell reorientation in response to uniaxial cyclic stretching. Cells (greyscale circle) retract immediately upon the release of stretching (red arrows), followed by progressive extension during the relaxation phase (blue arrows). The effects accumulate upon prolonged cyclic stretching, which leads to the change in cell shape and orientation.

In summary, the present results suggest that cyclic stretching induces two complementary events during the relaxation phase—an immediate retraction that decays after the first several cycles of stretching, and a slower but more persistent protrusion along the transverse direction. Reorientation perpendicular to the direction of stretching represents a cumulative result of stepwise axial retractions and transverse extension at each cycle (Fig. 7). In addition, microtubules may play a role in coordinating the activities between different regions, similar to their proposed role during directional cell migration.

## Materials and methods

### Cell culturing and pharmacological treatment

NRK-52E rat kidney epithelial cells (ATCC CRL-1571, Manassas, VA) were maintained at 37 °C with 5% (v/v) CO_2_ in Dulbecco's modified Eagle's medium (DMEM; Life Technologies, Carlsbad, CA) supplemented with 10% (v/v) fetal bovine serum (Thermo Scientific, Waltham, MA), 2 mM l-glutamine, 50 μg/ml streptomycin, and 50 units/ml penicillin (Life Technologies, Carlsbad, CA). Cells were plated on polyacrylamide (PAA) substrate of the stretcher for 36 h before stretching.

Cells were treated with 5 μM nocodazole (Sigma-Aldrich, St. Louis, MO) for 2 h or with 50 nM vincristine (Sigma-Aldrich, St. Louis, MO) for 20 h to induce microtubule depolymerization before the application of uniaxial cyclic stretching. Myosin-II contractility was inhibited by treating cells with 50 μM (–)-blebbistatin (Sigma-Aldrich, St. Louis, MO), prepared by slowly diluting a stock solution of 100 mM in DMSO into the warm medium under vigorous stirring followed by filtration with a 0.22 μm filter.

### Preparation of cell stretcher

The stretcher contained a sheet of PAA gel 7.5 mm × 5 mm × 0.4 mm in dimension, one end of which was bonded to a glass coverslip while the opposite end was attached to a handle for anchorage. To prepare the PAA gel, a glass coverslip (45 × 50 mm^2^ No. 2; Fisher Scientific) was activated at one end with Bind-Silane (GE Healthcare, Waukesha, WI) for bonding PAA and the remaining area was treated with water-repellant Rain‑X or Repel-Silane (GE Healthcare Life Science) to prevent the adhesion of PAA to the glass (Supplementary Fig. 1g). A casting chamber was then assembled with the activated coverslip, a handle made of a PDMS block attached to a bind-silane treated coverslip (Supplementary Fig. 1h), and a top coverslip micropatterned or coated with gelatin (Supplementary Fig. 1f,i,k)^49^. A solution of 10% acrylamide (Bio-Rad, Hercules, CA), 0.3% bis-acrylamide (Bio-Rad, Hercules, CA), 0.001% (w/v) ammonium persulfate (Sigma Aldrich, St. Louis, MO), and 0.004% (v/v) N,N,N′,N′-tetramethylene-1,2-diamine (TEMED, Bio-Rad, Hercules, CA) was injected into the chamber and allowed to polymerize for 45 min. Polymerization of acrylamide was initiated by the initiator ammonium persulfate in the mixture. The coverslip with PAA substrate was then attached with vacuum grease to an acrylic block with a hole, to form a culture chamber with a stretchable substrate (Fig. 1b).

Micropatterned coverslips were prepared as described previously^50^. To micropattern coverslips with gelatin, PDMS stamps were prepared by polymerizing Silgard 184 (Dow, Midland, MI) on a photoresist mold (Supplementary Fig. 1a,b; SPR 220, MicroChem, Round Rock, TX), and coated with 0.1%(w/v) periodate-activated gelatin (Supplementary Fig. 1c, 3.5 mg/ml sodium m-periodate, Sigma-Aldrich, St. Louis, MO), before pressing on a 25 × 25 mm^2^ No. 1.5 coverslip to transfer the pattern (Supplementary Fig. 1d,e). The coverslip was then used for covering the acrylamide solution during polymerization. The micropattern was revealed by 0.2 μm fluorescent latex beads (red, polystyrene; Molecular Probes, Carlsbad, CA) added to the acrylamide solution at a 1:1000 dilution, which became concentrated onto protein conjugated areas during acrylamide polymerization^50^. While a definitive mechanism is still lacking, we suspect that protein coating caused an increase in the binding affinity between glass and latex beads compared to bare glass surfaces.

### Strain analysis

Microcontact-printed squares 50 × 50 µm^2^ in area, as revealed by fluorescent latex beads that became concentrated onto protein-conjugated areas, were used for visualizing the strain of the PAA gel in response to the stress induced by stage movement. Deformation of the square pattern was imaged in response to stage movements calculated to stretch the gel by 5%, 10%, 15%, or 20%. Fluorescent images were analyzed with ImageJ (National Institutes of Health, Bethesda, MD), and the length and width of the square as a function of stretching distance was analyzed with linear regression. Residual strain upon relaxation was determined by measuring the offset of local marks before and after stretching.

### Microscopy and live-cell imaging

The microscope was covered with a plastic enclosure to serve as an incubator, wherein the temperature and CO_2_ concentration were maintained for cell viability. Phase-contrast images of cells were collected with a Zeiss Axiovet 200M microscope using a 40 × N.A. 0.55 phase contrast dry objective lens. The microscope was equipped with a high-precision motorized stage (MS-2000 XYZ, Applied Scientific Instrumentation, Eugene, OR). Before stretching, a rod on a micromanipulator was used for fixing the position of the handle attached to the PAA sheet, while the coverslip moved back and forth with the motorized stage. Custom software was used for controlling and coordinating stage movement and image acquisition.

### Analysis of cell orientation, cell length, and protrusion activities

Cell outline was drawn manually using ImageJ for determining the spreading area. The outline was then fit with an ellipse, and the orientation index was determined as *cos2θ*, where *θ* is the long axis of the ellipse. Perfect alignment parallel and perpendicular to the direction of stretching was indicated by an orientation index of 1 and − 1, respectively. Cell outline was also fitted with a rectangular to determine the cell transverse/axial length as the width/length of the rectangle. Protrusion and retraction activities were calculated using the difference of consecutive images, using a custom MATLAB program. Areas of net protrusion or retraction were normalized against the average cell spreading area.

### Traction force microscopy

For traction force microscopy, cells were cultured on a 10 kPa sheet of polyacrylamide gel, prepared and bonded to an activated glass coverslip as mentioned above^30,51^. The acrylamide solution was prepared with 10% acrylamide, 0.1% bis-acrylamide, 0.001% (w/v) ammonium persulfate, and 0.004% (v/v) N,N,N′,N′-tetramethylene-1,2-diamine and a 1:1000 dilution of 0.2 μm fluorescent latex beads. A 30 µl aliquot of this solution was pipetted onto a glass coverslip (43 × 50 mm^2^ No. 1; Fisher Scientific) activated with Bind-Silane as described above, then covered with a 25 × 25 mm^2^ No. 1.5 coverslip coated with activated gelatin. The top coverslip was removed after 30 min of polymerization.

Fluorescence images of beads near the top gel surface underneath a cell were acquired before and after nocodazole or blebbistatin treatment for 1 and 2 h. The fluorescent image of beads in relaxation positions was then acquired after removing the adherent cell by trypsinization. Displacements of the substrate as a result of traction forces were determined with particle image velocimetry, implemented as a plugin of ImageJ (https://sites.google.com/site/qingzongtseng/piv). Traction stresses were then calculated using Fourier transform traction cytometry (https://github.com/DanuserLab/TFM)^52,53^. Since significant traction stresses were found only under a small fraction of the cell area, changes in traction stress were determined based on 95 percentile stress.

### Statistics analysis

All the data were evaluated from at least three independent experiments conducted on different stretchers. Orientation index (*cos2θ*) was statistically analyzed by the Friedman test in conjunction with Dunn's multi-comparison test, since the data were non-normal distributed. Statistical significance of the difference in cell length at different points of a time sequence was determined by repeated one-way ANOVA, while the comparisons of cells under diverse conditions were conducted using simple *t*-test. Paired *t*-test was also used for determining the change in traction stress. Mixed model two-way ANOVA with Tukey's multi-comparisons test was used to determine the statistical difference of cell retraction following various periods of cyclic stretching.

## Supplementary Information

Supplementary Information.
