# Supplementary material for: Cyclic stretching-induced epithelial cell reorientation is driven by microtubule-modulated transverse extension during the relaxation phase
Source: Sci Rep. 2021 Jul 20;11:14803. doi: 10.1038/s41598-021-93987-y (PMC8292395; doi:10.1038/s41598-021-93987-y)
Supplement: Supplementary file 1 — Supplementary Information. [file 41598_2021_93987_MOESM1_ESM.pdf]

Supplementary Information for

Cyclic Stretching-Induced Epithelial Cell Reorientation is Driven by Microtubule-Modulated Transverse Extension during the Relaxation Phase

Jui-Chien Lien[a] and Yu-li Wang[a,*]

[a]Department of Biomedical Engineering, Carnegie Mellon University, Pittsburgh, PA 15213

Corresponding author: Yu-li Wang

Email: yuliwang@andrew.cmu.edu.

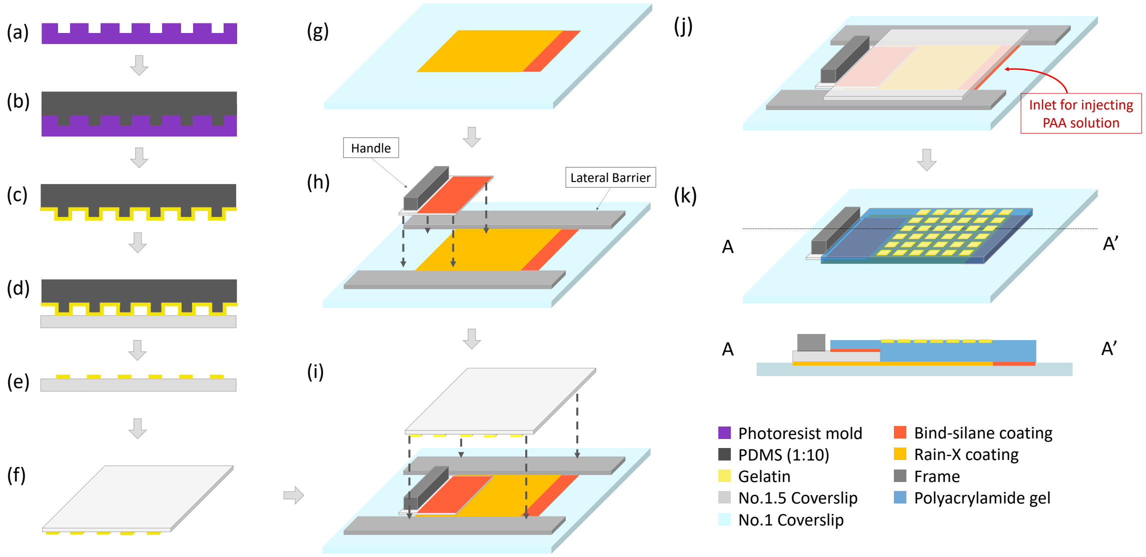

Supplementary Fig. 1. Fabrication of polyacrylamide stretcher. Photoresist prepared by photolithography (a) is used as a mold to fabricate PDMS stamp (b, gray). This stamp is removed from the photoresist mold and incubated with 0.1% gelatin (c, yellow) for the subsequent microcontact printing onto a coverslip (d-f), for the purpose of micropatterning the PAA surface (i). The coverslip for supporting the PAA gel is activated with bind-silane near one end (g, red) and treated with water-repellent Rain-X over the remaining area (g, orange). A PDMS handle is glued onto a trimmed piece of coverslip, which is then attached to the PAA gel using bind-silane (h). A casting chamber for PAA is formed using strips of coverslips, together with the handle and protein-printed top coverslip (i). Activated acrylamide solution is then injected into the chamber (j). After the completion of polymerization, the casting chamber is carefully disassembled to expose the PAA gel (k).

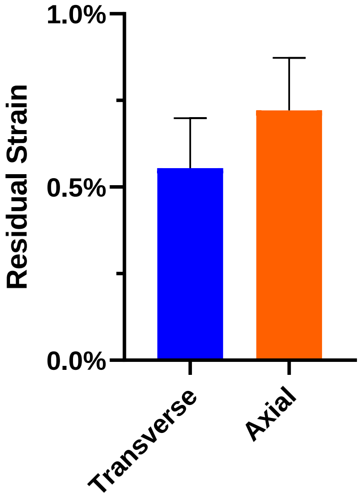

Supplementary Fig. 2. The residual strain of gel after cyclic stretching. The distances between local marks before/after stretching were used to determine the residual strain of the PAA gel. Residual strains in transverse and axial directions were both less than 0.8% of strain following 45 min of continuous cyclic stretching with 15% of strain at 0.5 Hz. (n = 20, Mean ± SEM.)

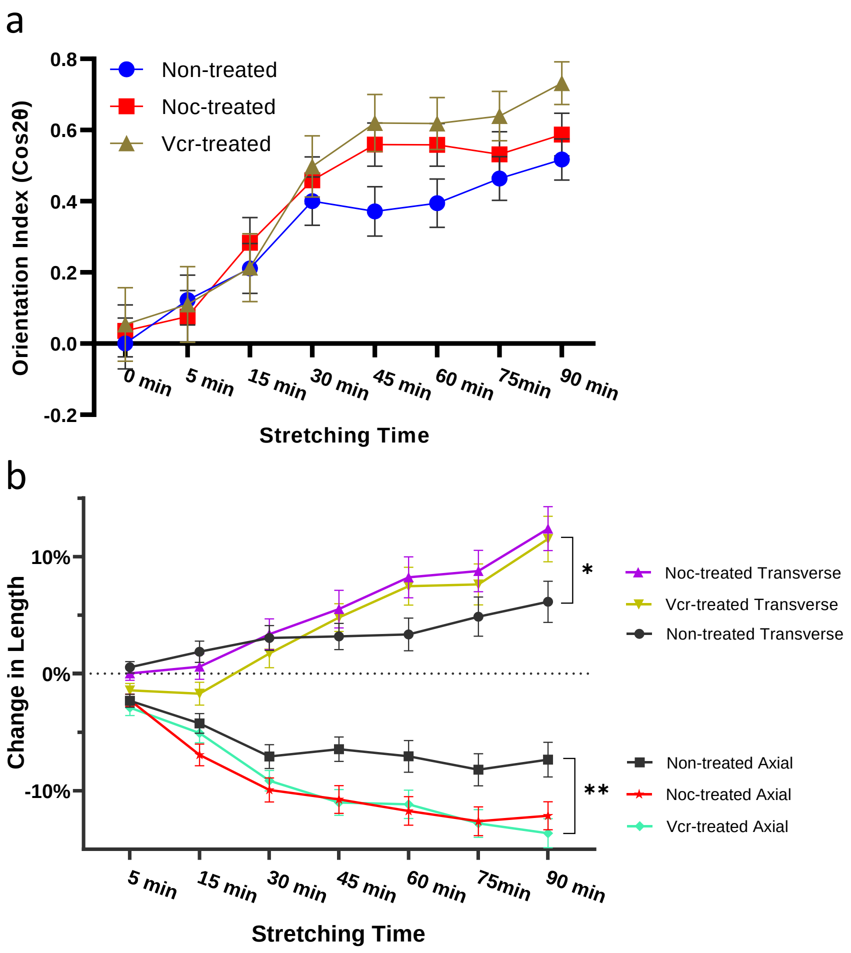

Supplementary Fig. 3 Effects of vincristine on cell reorientation in response to cyclic stretching. Cells are treated with 50 nM vincristine for 20 h before applying cyclic stretching. Reorientation is shown as changes in orientation index (a), or changes in axial and transverse lengths (b). The responses to vincristine (Vcr-treated) are similar to those after nocodazole treatment (noc-treated; using the same data as those shown in Fig. 6). Compared to the control, both transverse elongation and axial shortening are enhanced in cells treated with nocodazole or vincristine (b). (*, $p < 0.05$; **, $p < 0.01$; $n = 51$, Mean ± SEM).

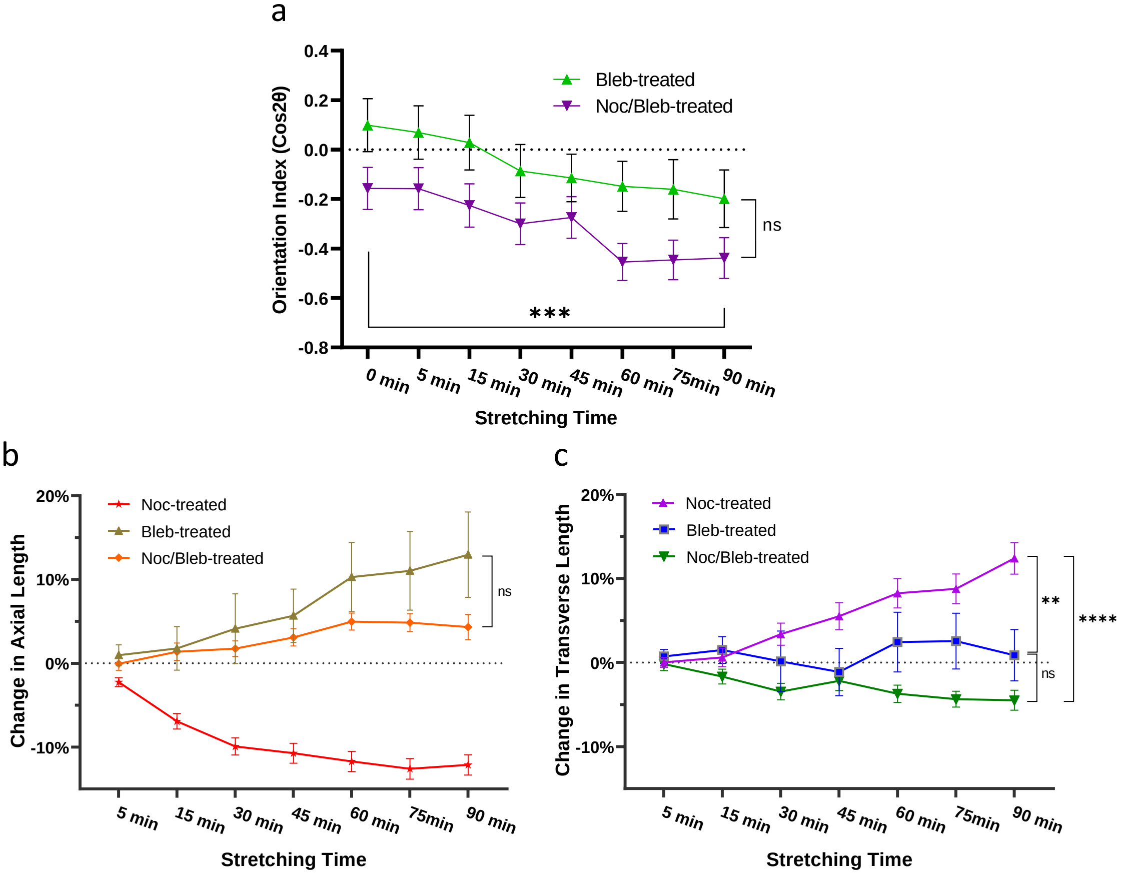

Supplementary Fig. 4. Combined effect of blebbistatin and nocodazole on cell reorientation in response to cyclic stretching. Myosin II activities and microtubule assembly are disrupted by treatment with 50 µM blebbistatin and 5 µM nocodazole for 2 h, before applying cyclic stretching. Treatment with blebbistatin with or without nocodazole causes cell reorientation along the direction of stretching (a), although treatment with both inhibitors induces a stronger effect. While cells treated with nocodazole alone show a decrease in axial length and increase in transferse length (b, c), cells treated with blebbistatin, with or without nocodazole, show the opposite response of increase in axial length and inhibition of the increase in transverse length. (n.s., $p > 0.05$; **, $p < 0.01$; ***, $p < 0.001$; ****, $p < 0.0001$; $n = 57$, Mean ± SEM.)

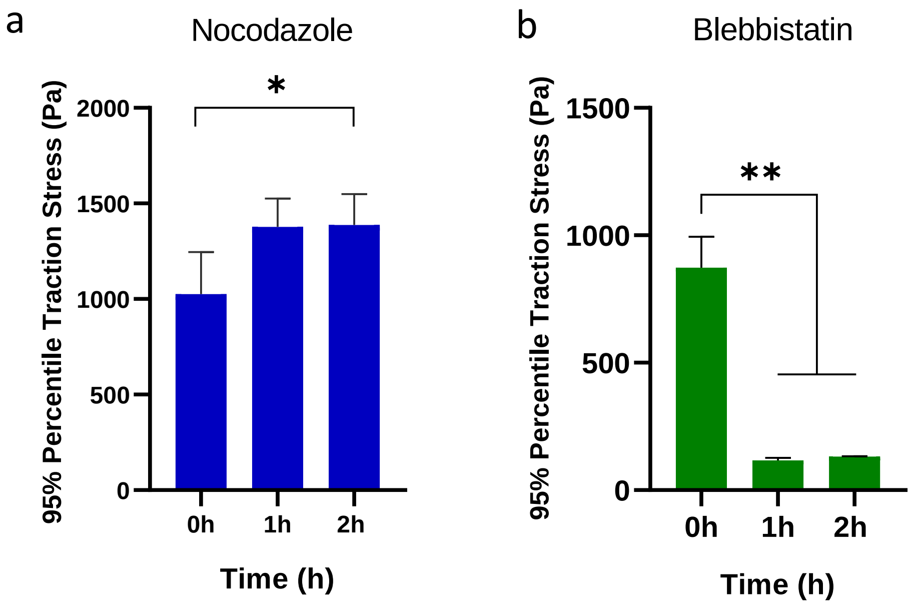

Supplementary Fig. 5. Changes of traction stress induced by nocodazole or blebbistatin. Treatment with 5 µM nocodazole induces an increase in traction stress by 30.4% within 1 hour (a), based on 95-percentile traction stress. In contrast, treatment with 50 µM blebbistatin causes a decrease of traction stress by 86.5% within 1 hour (b). (*, $p < 0.05$; **, $p < 0.01$; $n = 6$, Mean ± SEM.)

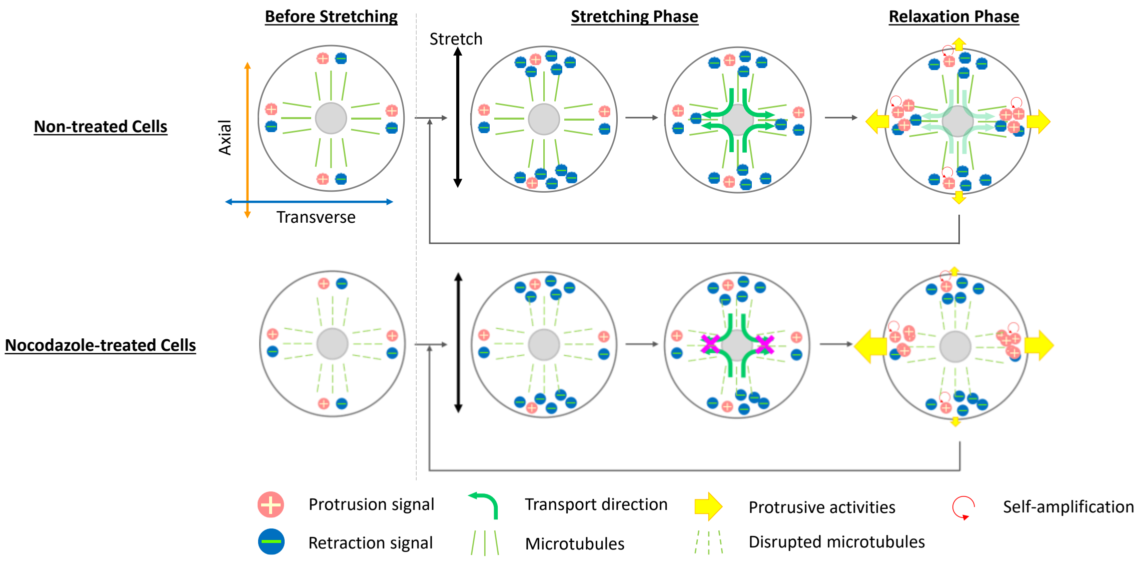

Supplementary Fig. 6. A possible mechanism for cyclic stretching-induced cell reorientation. Retraction signals are generated upon relaxation at axial ends, where they suppress local protrusion. These signals are transported by microtubules [1] to the transverse ends to modulate axial protrusions (upper panel). In nocodazole-treated cells (lower panel), the disassembly of microtubules causes retraction signals to accumulate at axial ends to enhance axial retraction. The corresponding decrease of retraction signals at transverse ends allows enhanced transverse protrusive activities.

Supplementary References

1 Zhang, J., Guo, W. H. & Wang, Y. L. Microtubules stabilize cell polarity by localizing rear signals. Proc Natl Acad Sci U S A 111, 16383-16388, doi:10.1073/pnas.1410533111 (2014).
